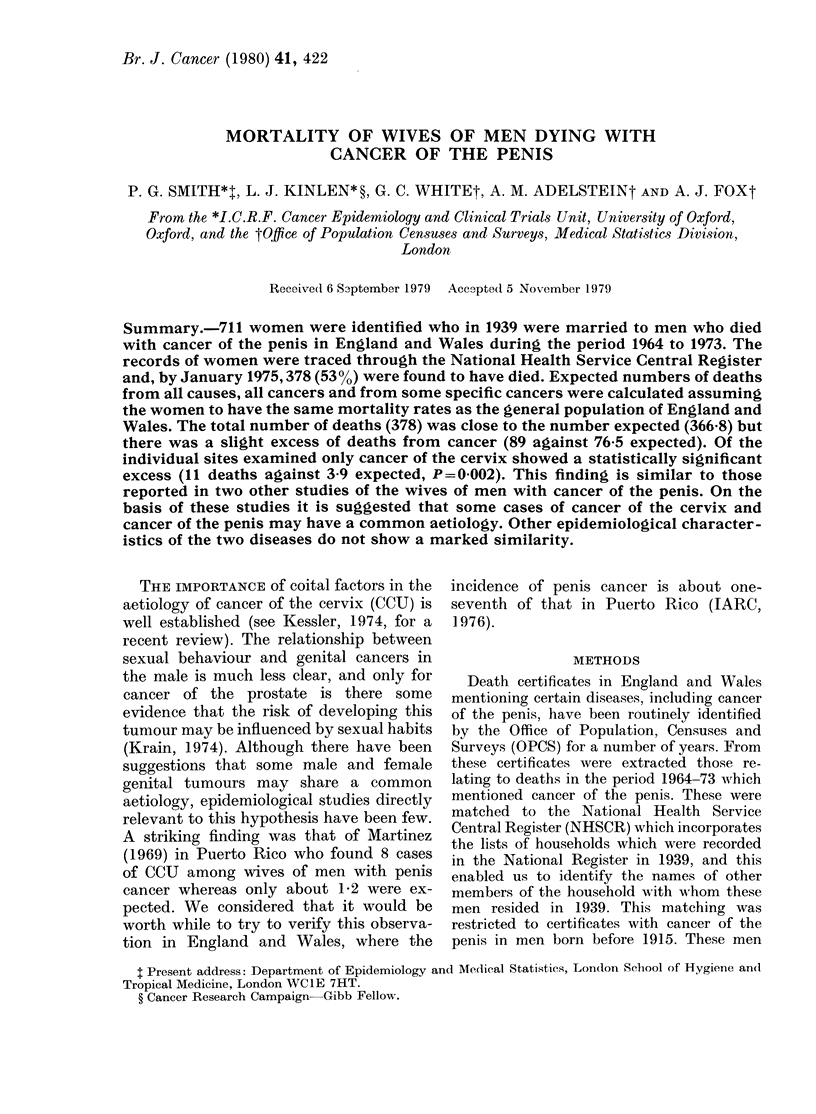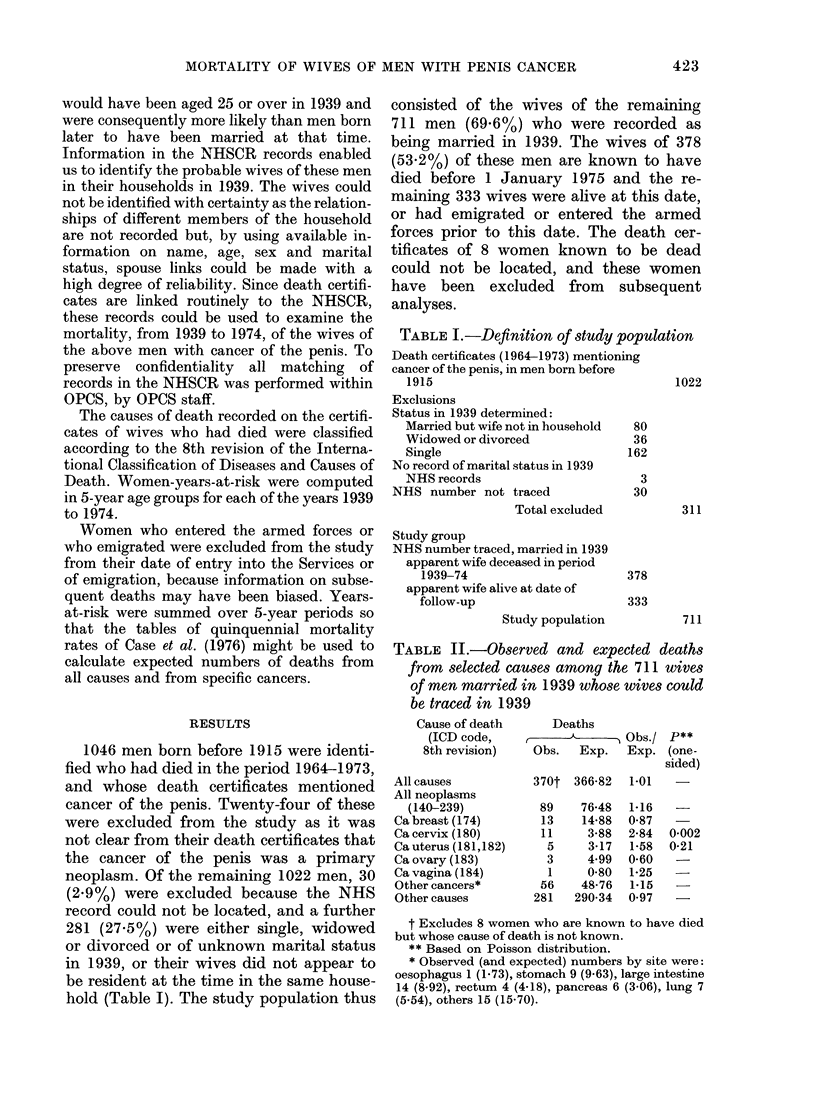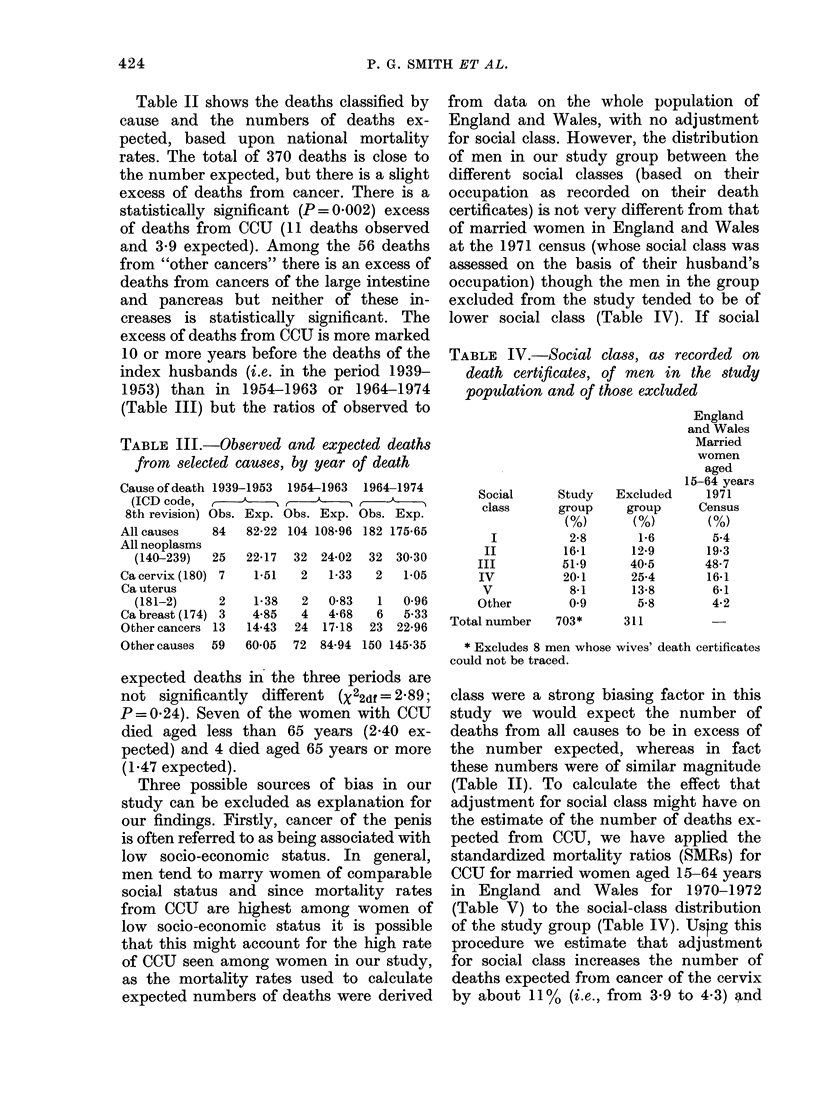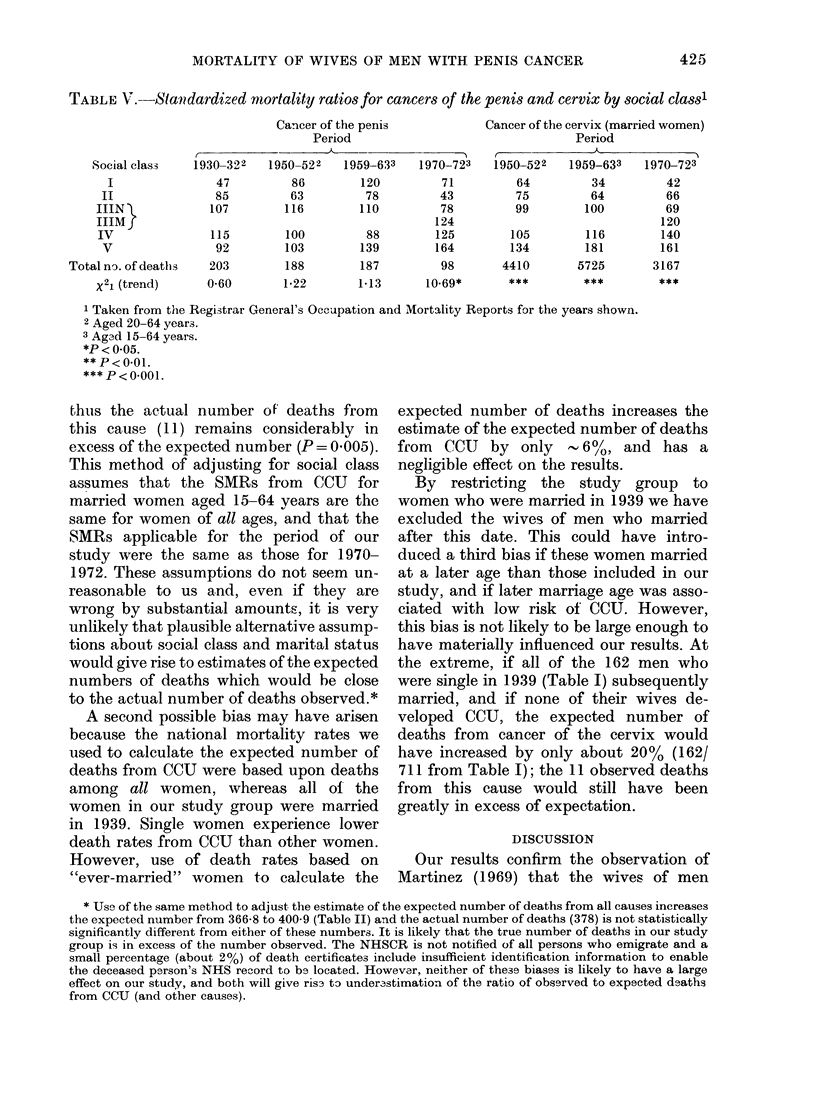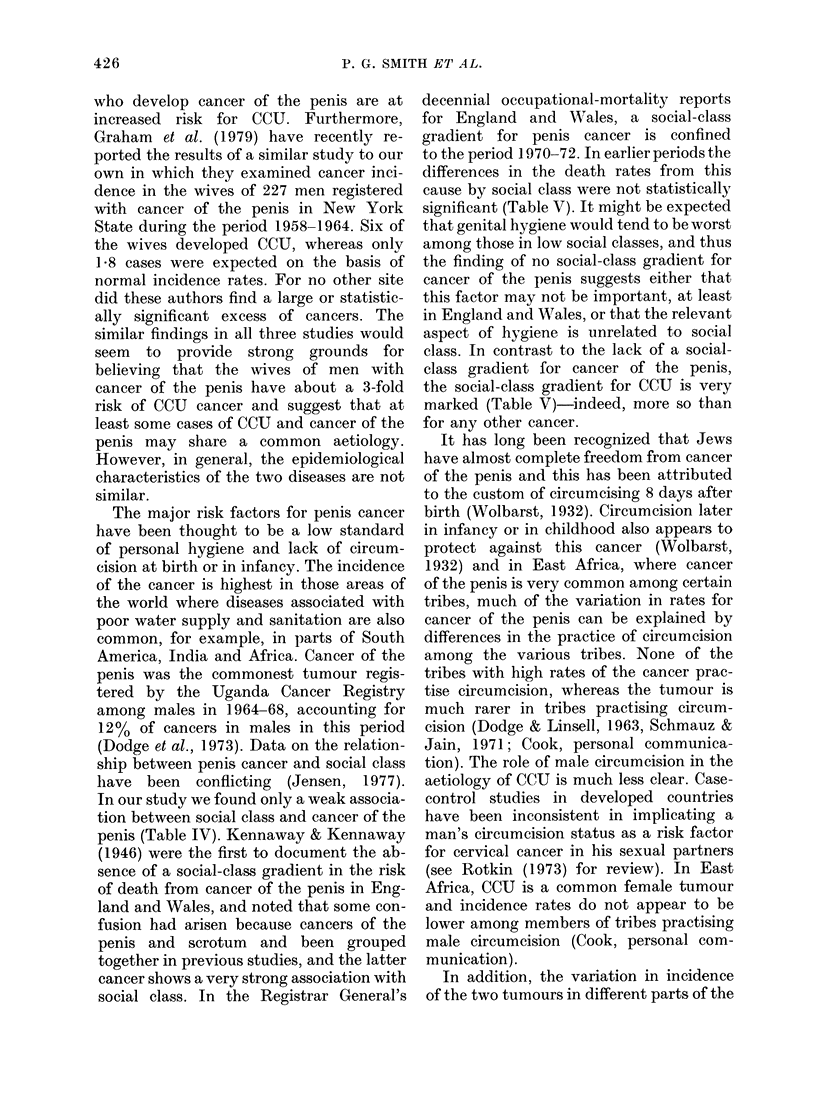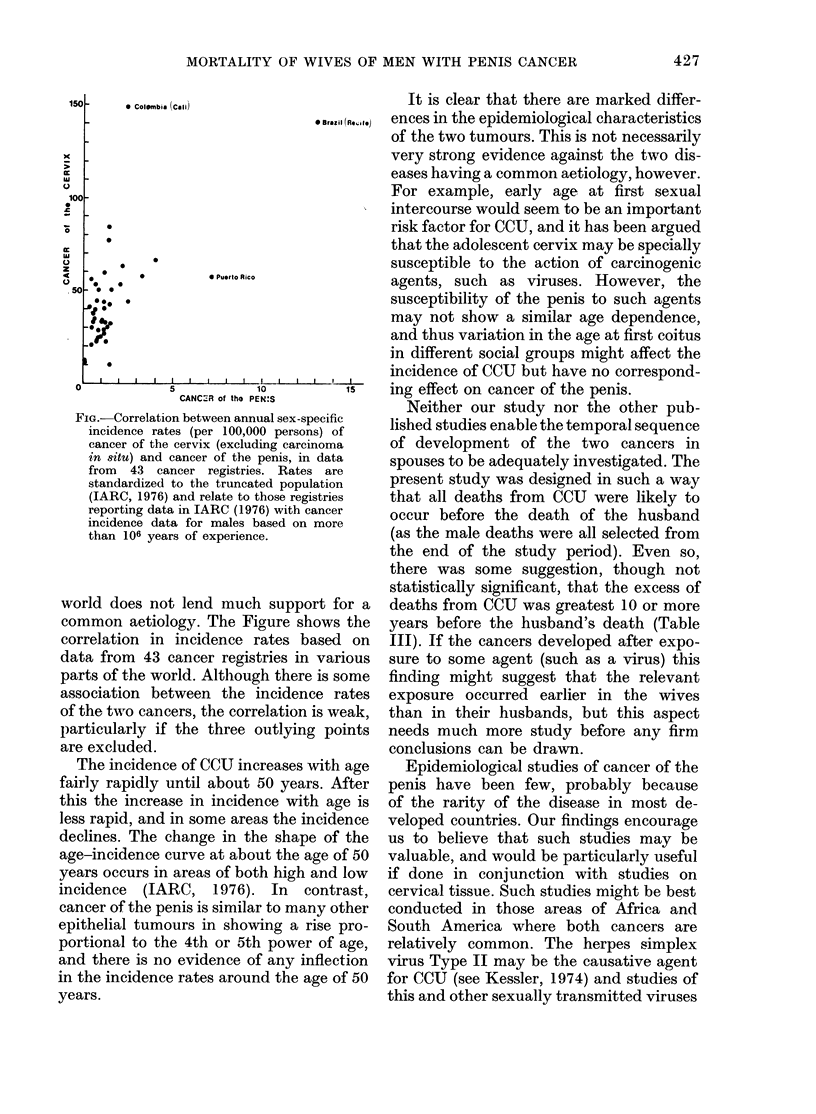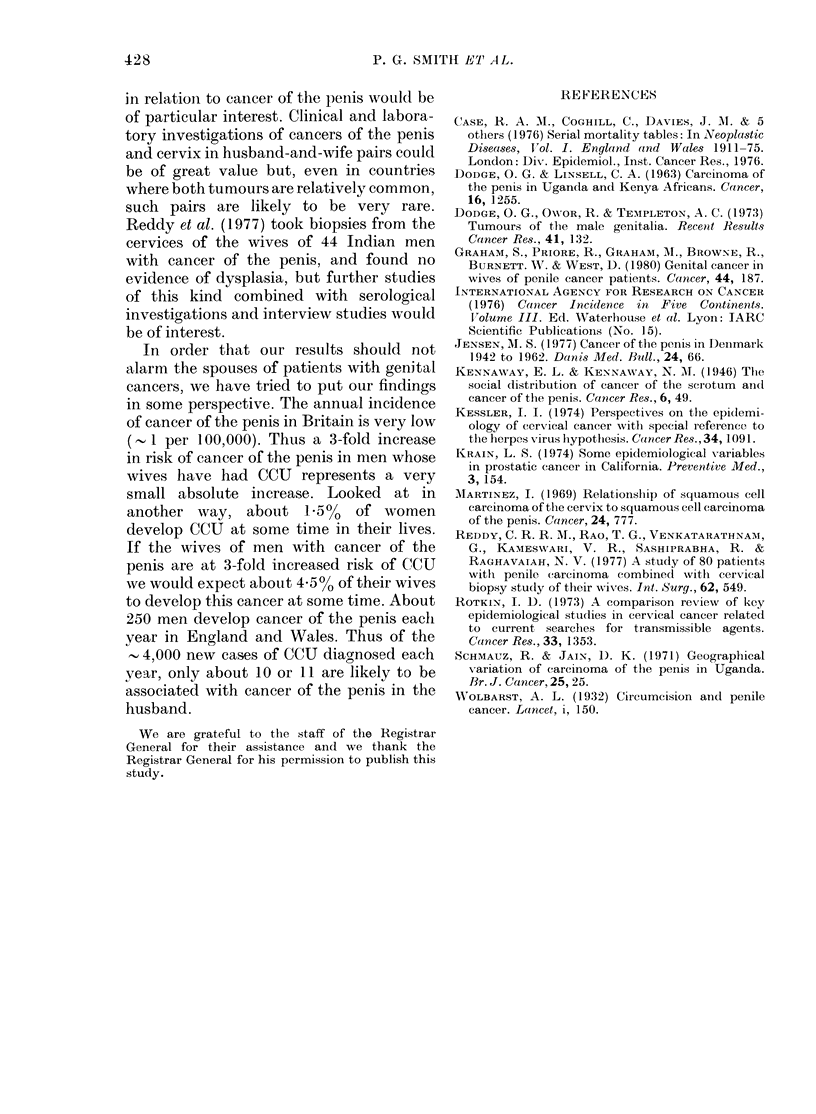# Mortality of wives of men dying with cancer of the penis.

**DOI:** 10.1038/bjc.1980.66

**Published:** 1980-03

**Authors:** P. G. Smith, L. J. Kinlen, G. C. White, A. M. Adelstein, A. J. Fox

## Abstract

711 women were identified who in 1939 were married to men who died with cancer of the penis in England and Wales during the period 1964 to 1973. The records of women were traced through the National Health Service Central Register and, by January 1975, 378 (53%) were found to have died. Expected numbers of deaths from all causes, all cancers and from some specific cancers were calculated assuming the women to have the same mortality rates as the general population of England and Wales. The total number of deaths (378) was close to the number expected (366-8) but there was a slight excess of deaths from cancer (89 against 76.5 expected). Of the individual sites examined only cancer of the cervix showed a statistically significant excess (11 deaths against 3.9 expected, P = 0.002). This finding is similar to those reported in two other studies of the wives of men with cancer of the penis. On the basis of these studies it is suggested that some cases of cancer of the cervix and cancer of the penis may have a common aetiology. Other epidemiological characteristics of the two diseases do not show a marked similarity.


*Br. J. Cancer* (1980) **41**, 422

### MORTALITY OF WIVES OF MEN DYING WITH CANCER OF THE PENIS

P. G. SMITH*‡, L. J. KINLEN*§, G. C. WHITE†, A. M. ADELSTEIN† AND A. J. FOX†

*From the *I.C.R.F. Cancer Epidemiology and Clinical Trials Unit, University of Oxford, Oxford, and the †Office of Population Censuses and Surveys, Medical Statistics Division, London*

Received 6 September 1979 Accepted 5 November 1979

**Summary.—711 women were identified who in 1939 were married to men who died with cancer of the penis in England and Wales during the period 1964 to 1973. The records of women were traced through the National Health Service Central Register and, by January 1975, 378 (53%) were found to have died. Expected numbers of deaths from all causes, all cancers and from some specific cancers were calculated assuming the women to have the same mortality rates as the general population of England and Wales. The total number of deaths (378) was close to the number expected (366·8) but there was a slight excess of deaths from cancer (89 against 76·5 expected). Of the individual sites examined only cancer of the cervix showed a statistically significant excess (11 deaths against 3·9 expected, $P=0{\cdot}002$). This finding is similar to those reported in two other studies of the wives of men with cancer of the penis. On the basis of these studies it is suggested that some cases of cancer of the cervix and cancer of the penis may have a common aetiology. Other epidemiological characteristics of the two diseases do not show a marked similarity.**

THE IMPORTANCE of coital factors in the aetiology of cancer of the cervix (CCU) is well established (see Kessler, 1974, for a recent review). The relationship between sexual behaviour and genital cancers in the male is much less clear, and only for cancer of the prostate is there some evidence that the risk of developing this tumour may be influenced by sexual habits (Krain, 1974). Although there have been suggestions that some male and female genital tumours may share a common aetiology, epidemiological studies directly relevant to this hypothesis have been few. A striking finding was that of Martinez (1969) in Puerto Rico who found 8 cases of CCU among wives of men with penis cancer whereas only about 1·2 were expected. We considered that it would be worth while to try to verify this observation in England and Wales, where the incidence of penis cancer is about one-seventh of that in Puerto Rico (IARC, 1976).

### METHODS

Death certificates in England and Wales mentioning certain diseases, including cancer of the penis, have been routinely identified by the Office of Population, Censuses and Surveys (OPCS) for a number of years. From these certificates were extracted those relating to deaths in the period 1964–73 which mentioned cancer of the penis. These were matched to the National Health Service Central Register (NHSCR) which incorporates the lists of households which were recorded in the National Register in 1939, and this enabled us to identify the names of other members of the household with whom these men resided in 1939. This matching was restricted to certificates with cancer of the penis in men born before 1915. These men

‡ Present address: Department of Epidemiology and Medical Statistics, London School of Hygiene and Tropical Medicine, London WC1E 7HT.
§ Cancer Research Campaign—Gibb Fellow.

would have been aged 25 or over in 1939 and were consequently more likely than men born later to have been married at that time. Information in the NHSCR records enabled us to identify the probable wives of these men in their households in 1939. The wives could not be identified with certainty as the relationships of different members of the household are not recorded but, by using available information on name, age, sex and marital status, spouse links could be made with a high degree of reliability. Since death certificates are linked routinely to the NHSCR, these records could be used to examine the mortality, from 1939 to 1974, of the wives of the above men with cancer of the penis. To preserve confidentiality all matching of records in the NHSCR was performed within OPCS, by OPCS staff.

The causes of death recorded on the certificates of wives who had died were classified according to the 8th revision of the International Classification of Diseases and Causes of Death. Women-years-at-risk were computed in 5-year age groups for each of the years 1939 to 1974.

Women who entered the armed forces or who emigrated were excluded from the study from their date of entry into the Services or of emigration, because information on subsequent deaths may have been biased. Years-at-risk were summed over 5-year periods so that the tables of quinquennial mortality rates of Case *et al.* (1976) might be used to calculate expected numbers of deaths from all causes and from specific cancers.

### RESULTS

1046 men born before 1915 were identified who had died in the period 1964–1973, and whose death certificates mentioned cancer of the penis. Twenty-four of these were excluded from the study as it was not clear from their death certificates that the cancer of the penis was a primary neoplasm. Of the remaining 1022 men, 30 (2·9%) were excluded because the NHS record could not be located, and a further 281 (27·5%) were either single, widowed or divorced or of unknown marital status in 1939, or their wives did not appear to be resident at the time in the same household (Table I). The study population thus consisted of the wives of the remaining 711 men (69·6%) who were recorded as being married in 1939. The wives of 378 (53·2%) of these men are known to have died before 1 January 1975 and the remaining 333 wives were alive at this date, or had emigrated or entered the armed forces prior to this date. The death certificates of 8 women known to be dead could not be located, and these women have been excluded from subsequent analyses.

TABLE I.—*Definition of study population*

| | | |
|---|---|---|
| Death certificates (1964–1973) mentioning cancer of the penis, in men born before 1915 | | 1022 |
| Exclusions | | |
| Status in 1939 determined: | | |
| Married but wife not in household | 80 | |
| Widowed or divorced | 36 | |
| Single | 162 | |
| No record of marital status in 1939 NHS records | 3 | |
| NHS number not traced | 30 | |
| Total excluded | | 311 |
| Study group | | |
| NHS number traced, married in 1939 | | |
| apparent wife deceased in period 1939–74 | 378 | |
| apparent wife alive at date of follow-up | 333 | |
| Study population | | 711 |

TABLE II.—*Observed and expected deaths from selected causes among the* 711 *wives of men married in* 1939 *whose wives could be traced in* 1939

| Cause of death (ICD code, 8th revision) | Deaths Obs. | Deaths Exp. | Obs./Exp. | $P$** (one-sided) |
|---|---|---|---|---|
| All causes | 370† | 366·82 | 1·01 | — |
| All neoplasms (140–239) | 89 | 76·48 | 1·16 | — |
| Ca breast (174) | 13 | 14·88 | 0·87 | — |
| Ca cervix (180) | 11 | 3·88 | 2·84 | 0·002 |
| Ca uterus (181,182) | 5 | 3·17 | 1·58 | 0·21 |
| Ca ovary (183) | 3 | 4·99 | 0·60 | — |
| Ca vagina (184) | 1 | 0·80 | 1·25 | — |
| Other cancers* | 56 | 48·76 | 1·15 | — |
| Other causes | 281 | 290·34 | 0·97 | — |

† Excludes 8 women who are known to have died but whose cause of death is not known.

** Based on Poisson distribution.

* Observed (and expected) numbers by site were: oesophagus 1 (1·73), stomach 9 (9·63), large intestine 14 (8·92), rectum 4 (4·18), pancreas 6 (3·06), lung 7 (5·54), others 15 (15·70).

Table II shows the deaths classified by cause and the numbers of deaths expected, based upon national mortality rates. The total of 370 deaths is close to the number expected, but there is a slight excess of deaths from cancer. There is a statistically significant ($P = 0{\cdot}002$) excess of deaths from CCU (11 deaths observed and 3·9 expected). Among the 56 deaths from "other cancers" there is an excess of deaths from cancers of the large intestine and pancreas but neither of these increases is statistically significant. The excess of deaths from CCU is more marked 10 or more years before the deaths of the index husbands (*i.e.* in the period 1939–1953) than in 1954–1963 or 1964–1974 (Table III) but the ratios of observed to

TABLE III.—*Observed and expected deaths from selected causes, by year of death*

| Cause of death (ICD code, 8th revision) | 1939–1953 Obs. | Exp. | 1954–1963 Obs. | Exp. | 1964–1974 Obs. | Exp. |
|---|---|---|---|---|---|---|
| All causes | 84 | 82·22 | 104 | 108·96 | 182 | 175·65 |
| All neoplasms (140–239) | 25 | 22·17 | 32 | 24·02 | 32 | 30·30 |
| Ca cervix (180) | 7 | 1·51 | 2 | 1·33 | 2 | 1·05 |
| Ca uterus (181–2) | 2 | 1·38 | 2 | 0·83 | 1 | 0·96 |
| Ca breast (174) | 3 | 4·85 | 4 | 4·68 | 6 | 5·33 |
| Other cancers | 13 | 14·43 | 24 | 17·18 | 23 | 22·96 |
| Other causes | 59 | 60·05 | 72 | 84·94 | 150 | 145·35 |

expected deaths in the three periods are not significantly different ($\chi^2_{2df} = 2{\cdot}89$; $P = 0{\cdot}24$). Seven of the women with CCU died aged less than 65 years (2·40 expected) and 4 died aged 65 years or more (1·47 expected).

Three possible sources of bias in our study can be excluded as explanation for our findings. Firstly, cancer of the penis is often referred to as being associated with low socio-economic status. In general, men tend to marry women of comparable social status and since mortality rates from CCU are highest among women of low socio-economic status it is possible that this might account for the high rate of CCU seen among women in our study, as the mortality rates used to calculate expected numbers of deaths were derived from data on the whole population of England and Wales, with no adjustment for social class. However, the distribution of men in our study group between the different social classes (based on their occupation as recorded on their death certificates) is not very different from that of married women in England and Wales at the 1971 census (whose social class was assessed on the basis of their husband's occupation) though the men in the group excluded from the study tended to be of lower social class (Table IV). If social

TABLE IV.—*Social class, as recorded on death certificates, of men in the study population and of those excluded*

| Social class | Study group (%) | Excluded group (%) | England and Wales Married women aged 15–64 years 1971 Census (%) |
|---|---|---|---|
| I | 2·8 | 1·6 | 5·4 |
| II | 16·1 | 12·9 | 19·3 |
| III | 51·9 | 40·5 | 48·7 |
| IV | 20·1 | 25·4 | 16·1 |
| V | 8·1 | 13·8 | 6·1 |
| Other | 0·9 | 5·8 | 4·2 |
| Total number | 703* | 311 | — |

* Excludes 8 men whose wives' death certificates could not be traced.

class were a strong biasing factor in this study we would expect the number of deaths from all causes to be in excess of the number expected, whereas in fact these numbers were of similar magnitude (Table II). To calculate the effect that adjustment for social class might have on the estimate of the number of deaths expected from CCU, we have applied the standardized mortality ratios (SMRs) for CCU for married women aged 15–64 years in England and Wales for 1970–1972 (Table V) to the social-class distribution of the study group (Table IV). Using this procedure we estimate that adjustment for social class increases the number of deaths expected from cancer of the cervix by about 11% (*i.e.*, from 3·9 to 4·3) and

TABLE V.—*Standardized mortality ratios for cancers of the penis and cervix by social class*[1]

| Social class | Cancer of the penis Period 1930–32[2] | 1950–52[2] | 1959–63[3] | 1970–72[3] | Cancer of the cervix (married women) Period 1950–52[2] | 1959–63[3] | 1970–72[3] |
|---|---|---|---|---|---|---|---|
| I | 47 | 86 | 120 | 71 | 64 | 34 | 42 |
| II | 85 | 63 | 78 | 43 | 75 | 64 | 66 |
| IIIN } | 107 | 116 | 110 | 78 | 99 | 100 | 69 |
| IIIM } | | | | 124 | | | 120 |
| IV | 115 | 100 | 88 | 125 | 105 | 116 | 140 |
| V | 92 | 103 | 139 | 164 | 134 | 181 | 161 |
| Total no. of deaths | 203 | 188 | 187 | 98 | 4410 | 5725 | 3167 |
| $\chi^2_1$ (trend) | 0·60 | 1·22 | 1·13 | 10·69* | *** | *** | *** |

[1] Taken from the Registrar General's Occupation and Mortality Reports for the years shown.
[2] Aged 20–64 years.
[3] Aged 15–64 years.
*$P < 0{\cdot}05$.
** $P < 0{\cdot}01$.
*** $P < 0{\cdot}001$.

thus the actual number of deaths from this cause (11) remains considerably in excess of the expected number ($P = 0{\cdot}005$). This method of adjusting for social class assumes that the SMRs from CCU for married women aged 15–64 years are the same for women of *all* ages, and that the SMRs applicable for the period of our study were the same as those for 1970–1972. These assumptions do not seem unreasonable to us and, even if they are wrong by substantial amounts, it is very unlikely that plausible alternative assumptions about social class and marital status would give rise to estimates of the expected numbers of deaths which would be close to the actual number of deaths observed.*

A second possible bias may have arisen because the national mortality rates we used to calculate the expected number of deaths from CCU were based upon deaths among *all* women, whereas all of the women in our study group were married in 1939. Single women experience lower death rates from CCU than other women. However, use of death rates based on "ever-married" women to calculate the expected number of deaths increases the estimate of the expected number of deaths from CCU by only ~6%, and has a negligible effect on the results.

By restricting the study group to women who were married in 1939 we have excluded the wives of men who married after this date. This could have introduced a third bias if these women married at a later age than those included in our study, and if later marriage age was associated with low risk of CCU. However, this bias is not likely to be large enough to have materially influenced our results. At the extreme, if all of the 162 men who were single in 1939 (Table I) subsequently married, and if none of their wives developed CCU, the expected number of deaths from cancer of the cervix would have increased by only about 20% (162/711 from Table I); the 11 observed deaths from this cause would still have been greatly in excess of expectation.

### DISCUSSION

Our results confirm the observation of Martinez (1969) that the wives of men

* Use of the same method to adjust the estimate of the expected number of deaths from all causes increases the expected number from 366·8 to 400·9 (Table II) and the actual number of deaths (378) is not statistically significantly different from either of these numbers. It is likely that the true number of deaths in our study group is in excess of the number observed. The NHSCR is not notified of all persons who emigrate and a small percentage (about 2%) of death certificates include insufficient identification information to enable the deceased person's NHS record to be located. However, neither of these biases is likely to have a large effect on our study, and both will give rise to underestimation of the ratio of observed to expected deaths from CCU (and other causes).

who develop cancer of the penis are at increased risk for CCU. Furthermore, Graham *et al.* (1979) have recently reported the results of a similar study to our own in which they examined cancer incidence in the wives of 227 men registered with cancer of the penis in New York State during the period 1958–1964. Six of the wives developed CCU, whereas only 1·8 cases were expected on the basis of normal incidence rates. For no other site did these authors find a large or statistically significant excess of cancers. The similar findings in all three studies would seem to provide strong grounds for believing that the wives of men with cancer of the penis have about a 3-fold risk of CCU cancer and suggest that at least some cases of CCU and cancer of the penis may share a common aetiology. However, in general, the epidemiological characteristics of the two diseases are not similar.

The major risk factors for penis cancer have been thought to be a low standard of personal hygiene and lack of circumcision at birth or in infancy. The incidence of the cancer is highest in those areas of the world where diseases associated with poor water supply and sanitation are also common, for example, in parts of South America, India and Africa. Cancer of the penis was the commonest tumour registered by the Uganda Cancer Registry among males in 1964–68, accounting for 12% of cancers in males in this period (Dodge *et al.*, 1973). Data on the relationship between penis cancer and social class have been conflicting (Jensen, 1977). In our study we found only a weak association between social class and cancer of the penis (Table IV). Kennaway & Kennaway (1946) were the first to document the absence of a social-class gradient in the risk of death from cancer of the penis in England and Wales, and noted that some confusion had arisen because cancers of the penis and scrotum and been grouped together in previous studies, and the latter cancer shows a very strong association with social class. In the Registrar General's decennial occupational-mortality reports for England and Wales, a social-class gradient for penis cancer is confined to the period 1970–72. In earlier periods the differences in the death rates from this cause by social class were not statistically significant (Table V). It might be expected that genital hygiene would tend to be worst among those in low social classes, and thus the finding of no social-class gradient for cancer of the penis suggests either that this factor may not be important, at least in England and Wales, or that the relevant aspect of hygiene is unrelated to social class. In contrast to the lack of a social-class gradient for cancer of the penis, the social-class gradient for CCU is very marked (Table V)—indeed, more so than for any other cancer.

It has long been recognized that Jews have almost complete freedom from cancer of the penis and this has been attributed to the custom of circumcising 8 days after birth (Wolbarst, 1932). Circumcision later in infancy or in childhood also appears to protect against this cancer (Wolbarst, 1932) and in East Africa, where cancer of the penis is very common among certain tribes, much of the variation in rates for cancer of the penis can be explained by differences in the practice of circumcision among the various tribes. None of the tribes with high rates of the cancer practise circumcision, whereas the tumour is much rarer in tribes practising circumcision (Dodge & Linsell, 1963, Schmauz & Jain, 1971; Cook, personal communication). The role of male circumcision in the aetiology of CCU is much less clear. Case-control studies in developed countries have been inconsistent in implicating a man's circumcision status as a risk factor for cervical cancer in his sexual partners (see Rotkin (1973) for review). In East Africa, CCU is a common female tumour and incidence rates do not appear to be lower among members of tribes practising male circumcision (Cook, personal communication).

In addition, the variation in incidence of the two tumours in different parts of the

FIG.—Correlation between annual sex-specific incidence rates (per 100,000 persons) of cancer of the cervix (excluding carcinoma *in situ*) and cancer of the penis, in data from 43 cancer registries. Rates are standardized to the truncated population (IARC, 1976) and relate to those registries reporting data in IARC (1976) with cancer incidence data for males based on more than $10^6$ years of experience.

world does not lend much support for a common aetiology. The Figure shows the correlation in incidence rates based on data from 43 cancer registries in various parts of the world. Although there is some association between the incidence rates of the two cancers, the correlation is weak, particularly if the three outlying points are excluded.

The incidence of CCU increases with age fairly rapidly until about 50 years. After this the increase in incidence with age is less rapid, and in some areas the incidence declines. The change in the shape of the age–incidence curve at about the age of 50 years occurs in areas of both high and low incidence (IARC, 1976). In contrast, cancer of the penis is similar to many other epithelial tumours in showing a rise proportional to the 4th or 5th power of age, and there is no evidence of any inflection in the incidence rates around the age of 50 years.

It is clear that there are marked differences in the epidemiological characteristics of the two tumours. This is not necessarily very strong evidence against the two diseases having a common aetiology, however. For example, early age at first sexual intercourse would seem to be an important risk factor for CCU, and it has been argued that the adolescent cervix may be specially susceptible to the action of carcinogenic agents, such as viruses. However, the susceptibility of the penis to such agents may not show a similar age dependence, and thus variation in the age at first coitus in different social groups might affect the incidence of CCU but have no corresponding effect on cancer of the penis.

Neither our study nor the other published studies enable the temporal sequence of development of the two cancers in spouses to be adequately investigated. The present study was designed in such a way that all deaths from CCU were likely to occur before the death of the husband (as the male deaths were all selected from the end of the study period). Even so, there was some suggestion, though not statistically significant, that the excess of deaths from CCU was greatest 10 or more years before the husband's death (Table III). If the cancers developed after exposure to some agent (such as a virus) this finding might suggest that the relevant exposure occurred earlier in the wives than in their husbands, but this aspect needs much more study before any firm conclusions can be drawn.

Epidemiological studies of cancer of the penis have been few, probably because of the rarity of the disease in most developed countries. Our findings encourage us to believe that such studies may be valuable, and would be particularly useful if done in conjunction with studies on cervical tissue. Such studies might be best conducted in those areas of Africa and South America where both cancers are relatively common. The herpes simplex virus Type II may be the causative agent for CCU (see Kessler, 1974) and studies of this and other sexually transmitted viruses in relation to cancer of the penis would be of particular interest. Clinical and laboratory investigations of cancers of the penis and cervix in husband-and-wife pairs could be of great value but, even in countries where both tumours are relatively common, such pairs are likely to be very rare. Reddy *et al.* (1977) took biopsies from the cervices of the wives of 44 Indian men with cancer of the penis, and found no evidence of dysplasia, but further studies of this kind combined with serological investigations and interview studies would be of interest.

In order that our results should not alarm the spouses of patients with genital cancers, we have tried to put our findings in some perspective. The annual incidence of cancer of the penis in Britain is very low ($\sim$1 per 100,000). Thus a 3-fold increase in risk of cancer of the penis in men whose wives have had CCU represents a very small absolute increase. Looked at in another way, about 1·5% of women develop CCU at some time in their lives. If the wives of men with cancer of the penis are at 3-fold increased risk of CCU we would expect about 4·5% of their wives to develop this cancer at some time. About 250 men develop cancer of the penis each year in England and Wales. Thus of the $\sim$4,000 new cases of CCU diagnosed each year, only about 10 or 11 are likely to be associated with cancer of the penis in the husband.

We are grateful to the staff of the Registrar General for their assistance and we thank the Registrar General for his permission to publish this study.

REFERENCES

CASE, R. A. M., COGHILL, C., DAVIES, J. M. & 5 others (1976) Serial mortality tables: In *Neoplastic Diseases, Vol. I. England and Wales* 1911–75. London: Div. Epidemiol., Inst. Cancer Res., 1976.

DODGE, O. G. & LINSELL, C. A. (1963) Carcinoma of the penis in Uganda and Kenya Africans. *Cancer*, **16**, 1255.

DODGE, O. G., OWOR, R. & TEMPLETON, A. C. (1973) Tumours of the male genitalia. *Recent Results Cancer Res.*, **41**, 132.

GRAHAM, S., PRIORE, R., GRAHAM, M., BROWNE, R., BURNETT. W. & WEST, D. (1980) Genital cancer in wives of penile cancer patients. *Cancer*, **44**, 187.

INTERNATIONAL AGENCY FOR RESEARCH ON CANCER (1976) *Cancer Incidence in Five Continents. Volume III*. Ed. Waterhouse *et al.* Lyon: IARC Scientific Publications (No. 15).

JENSEN, M. S. (1977) Cancer of the penis in Denmark 1942 to 1962. *Danis Med. Bull.*, **24**, 66.

KENNAWAY, E. L. & KENNAWAY, N. M. (1946) The social distribution of cancer of the scrotum and cancer of the penis. *Cancer Res.*, **6**, 49.

KESSLER, I. I. (1974) Perspectives on the epidemiology of cervical cancer with special reference to the herpes virus hypothesis. *Cancer Res.*, **34**, 1091.

KRAIN, L. S. (1974) Some epidemiological variables in prostatic cancer in California. *Preventive Med.*, **3**, 154.

MARTINEZ, I. (1969) Relationship of squamous cell carcinoma of the cervix to squamous cell carcinoma of the penis. *Cancer*, **24**, 777.

REDDY, C. R. R. M., RAO, T. G., VENKATARATHNAM, G., KAMESWARI, V. R., SASHIPRABHA, R. & RAGHAVAIAH, N. V. (1977) A study of 80 patients with penile carcinoma combined with cervical biopsy study of their wives. *Int. Surg.*, **62**, 549.

ROTKIN, I. D. (1973) A comparison review of key epidemiological studies in cervical cancer related to current searches for transmissible agents. *Cancer Res.*, **33**, 1353.

SCHMAUZ, R. & JAIN, D. K. (1971) Geographical variation of carcinoma of the penis in Uganda. *Br. J. Cancer*, **25**, 25.

WOLBARST, A. L. (1932) Circumcision and penile cancer. *Lancet*, i, 150.